# Flavonoids as CDK1 Inhibitors: Insights in Their Binding Orientations and Structure-Activity Relationship

**DOI:** 10.1371/journal.pone.0161111

**Published:** 2016-08-12

**Authors:** Carlos Navarro-Retamal, Julio Caballero

**Affiliations:** Centro de Bioinformática y Simulación Molecular, Facultad de Ingeniería, Universidad de Talca, 2 Norte 685, Casilla 721, Talca, Chile; University of Pittsburgh School of Medicine, UNITED STATES

## Abstract

In the last years, the interactions of flavonoids with protein kinases (PKs) have been described by using crystallographic experiments. Interestingly, different orientations have been found for one flavonoid inside different PKs and different chemical substitutions lead to different orientations of the flavonoid scaffold inside one PK. Accordingly, orientation predictions of novel analogues could help to the design of flavonoids with high PK inhibitory activities. With this in mind, we studied the binding modes of 37 flavonoids (flavones and chalcones) inside the cyclin-dependent PK CDK1 using docking experiments. We found that the compounds under study adopted two different orientations into the active site of CDK1 (orientations I and II in the manuscript). In addition, quantitative structure–activity relationship (QSAR) models using CoMFA and CoMSIA methodologies were constructed to explain the trend of the CDK1 inhibitory activities for the studied flavonoids. Template-based and docking-based alignments were used. Models developed starting from docking-based alignment were applied for describing the whole dataset and compounds with orientation I. Adequate R^2^ and Q^2^ values were obtained by each method; interestingly, only hydrophobic and hydrogen bond donor fields describe the differential potency of the flavonoids as CDK1 inhibitors for both defined alignments and subsets. Our current application of docking and QSAR together reveals important elements to be drawn for the design of novel flavonoids with increased PK inhibitory activities.

## Introduction

Flavonoids, natural products found abundantly in vegetables and fruits, are phytonutrients with many positive health benefits for humans [1]. They are famous for their antioxidant and anti-inflammatory health benefits, as well as their contribution of flashy color to the foods we eat; they also provide benefits in the prevention of chronic diseases such as diabetes, osteoporosis and cancer caused by free-radical damage [2–5].

In recent literature, naturally occurring and synthesized flavonoids has been identified as protein kinase (PK) inhibitors, targets associated to many of the processes related to the above mentioned diseases [6–8]. For instance, recent reports have revealed that flavonoids act at PK signaling pathways [9,10]. Specifically, flavonoids bind directly to some PKs, such as phosphoinositide 3-kinase (PI3K) [11], Akt/protein kinase B (Akt/PKB) [12], protein kinase C (PKC) [13], and mitogen-activated protein kinase (MAPKs) [14]. When interacting, flavonoids alter PK phosphorylation state to regulate multiple cell signaling pathways. This process has been associated to mechanism for the antioxidant functions of flavonoids, since they can exert their antioxidant properties through binding PKs to regulate the expression of antioxidant enzymes [15,16].

CDK1 is a cyclin-dependent kinase (CDK), a family of PKs, which play a key role in regulation of the cell cycle [17]. CDKs depend on regulatory subunits named cyclin, and their activities are modulated by CDK inhibitory proteins (CDKIPs). In many human cancers, such as melanomas, CDKs are overexpressed or CDKIPs are either absent or mutated. Therefore, CDKs have become attractive therapeutic targets to prevent unregulated proliferation of cancer cells. Consequently, in the last decades selective CDK inhibitors have been designed and evaluated as effective chemotherapeutic agents.

CDK1 is an essential member in the CDKs family required for successful completion of M-phase[18]. CDK1 is also the only CDK that can form complex with cyclin B, which start to accumulate at S-phase[19]. CDK1/cyclin B complex starts mitosis phase, while both, CDK1/Cyclin A and CDK1/Cyclin B are needed for mitosis to complete successfully[20–22].

In a recent report, series of flavonoids, specifically flavones and chalcones containing nitrogen, have been reported as CDK1 inhibitors [23,24]. These compounds are based on flavopiridol, which induce cell-cycle arrest at both G1 and G2 phases, and is a potent ATP competitive inhibitor of CDK1, 2, 4, and 6. In this work, the structural characteristics of the complexes between CDK1 and these compounds were elucidated by using a molecular modeling protocol based in docking. As a result, atomistic models of the active conformations were proposed and the interactions that contribute to form the complexes were discussed. Quantitative structure–activity relationship (QSAR) models were also developed using CoMFA and CoMSIA methods; the quality of such models was demonstrated by using predictive statistics. Together, docking-QSAR methodology provide novel information about the interactions between flavonoids and PKs that complement the information provided by crystallographic experiments and wet medicinal chemistry.

## Materials and Methods

### Modeling of flavonoid structures

**Fig 1:**
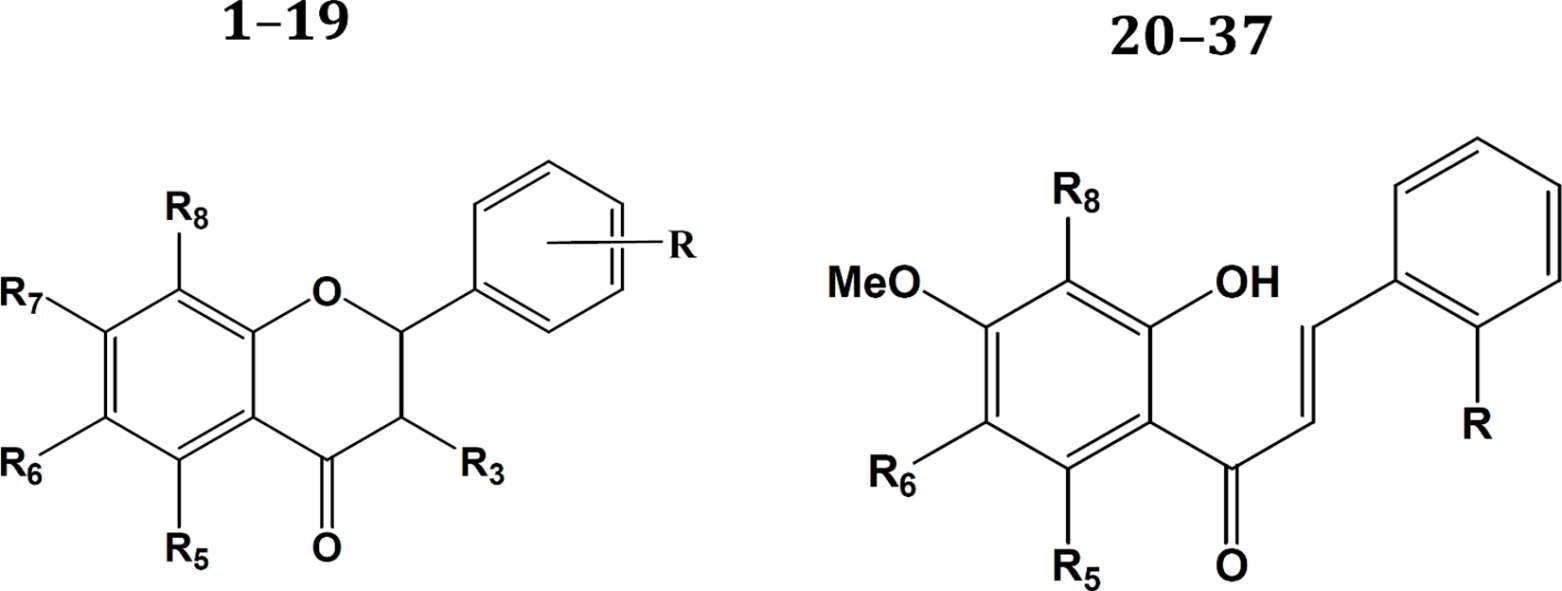
Structures of flavones (1–19) and chalcones (20–37).

The set of flavones and chalcones used in this study and their CDK1 inhibitory activities were collected from the articles of Liu et al. [24] and Zhang et al. [23]. The structures were sketched using Maestro’s molecular editor (Maestro 10.2.011, Schrödinger LLC). The biological activities of the compounds were converted to 1/log(IC_50_), where IC_50_ values represent the inhibitory amount (μM) to inhibit the 50% of the CDK1 enzymatic activity. All compounds and their respective activities are summarized in Fig 1, Table 1 and Table 2.

**Table 1:** Structures of flavones as CDK1 inhibitors. Experimental and predicted activities (log(1/IC_50_)) using models CoMSIA models.

Compounds^a^	R	R_3_	R_5_	R_6_	R_7_	R_8_	Log(1/IC_50_)			
							exp	predicted		
								SA-CoMSIA-HD	DAC2-CoMSIA-SHD	DAAC-CoMSIA-HD
**1**^b^	2 Cl	H	OMe	H	OMe	(morpholin-4-yl)Me	-0.477	0.426	0.468	0.677
**2**	2 Cl	H	OMe	H	OMe	(piperidin-1-yl)Me	0.811	0.247	0.692	0.924
**3**	2 Cl	H	OH	H	OMe	(morpholin-4-yl)Me	-0.477	0.453	0.199	0.314
**4**	2 Cl	H	OH	H	OMe	(piperidin-1-yl)Me	0.859	0.066	0.404	0.477
**5** (baicalin)	H	H	OH	OH	O-Glucuronide	H	-1.157	-1.011	-1.080	-1.251
**6**^b^	H	H	OH	OH	OH	H	-0.815	-0.100	-0.276	-0.122
**7**	H	H	OH	OH	OH	(dimethylamino)Me	-0.021	0.198	-0.043	-0.130
**8**	H	H	OH	OH	OH	(pyrrolidin-1-yl)Me	-0.086	0.400	0.264	0.353
**9**^b^	H	H	OH	OH	OH	(piperidin-1-yl)Me	-0.107	0.392	0.289	0.398
**10**	H	H	OH	OH	OH	(morpholin-4-yl)Me	0.553	0.193	0.074	0.202
**11**	H	H	OH	OH	OH	(thiomorpholin-4-yl)Me	0.420	0.284	0.140	0.239
**12**^b^	H	H	OH	OH	OH	(4-methylpiperazin-1-yl)Me	0.569	0.305	0.034	0.079
**13** (methyl ester of baicalin)	H	H	OH	OH	O-Glucuronide methyl ester	H	-1.066	-1.104	-0.969	-1.126
**14**	3,4 diOH	OH	OH	H	OH	H	-1.301	-1.764	-1.484	-1.452
**15**	3,4 diOH	OH	OH	H	OH	(dimethylamino)Me	-1.197	-1.278	-1.054	-1.203
**16**	3,4 diOH	OH	OH	H	OH	(piperidin-1-yl)Me	-1.272	-0.977	-1.280	-1.258
**17**	3,4 diOH	OH	OH	H	OH	(morpholin-4-yl)Me	-1.301	-1.063	-1.346	-1.382
**18**	3,4 diOH	OH	OH	H	OH	(4-methylpiperazin-1-yl)Me	-1.301	-1.085	-1.103	-1.098
**19 (flavopiridol)**	2 Cl	H	OH	H	OH	3-OH-1-methylpiperidin-4-yl	0.481	-0.078	0.633	0.592

^a^Compounds **1**–**4** are from reference [24], compounds 5–19 are from reference [23].

^b^Compounds predicted in the test set.

**Table 2:** Structures of chalcones as CDK1 inhibitors. Experimental and predicted activities (log(1/IC_50_)) using models CoMSIA models.

Compounds^a^	R	R_5_	R_6_	R_8_	Log(1/IC_50_)			
					exp	predicted		
						SA-CoMSIA-HD	DAC2-CoMSIA-SHD	DAAC-CoMSIA-HD
**20**	Cl	OMe	H	(morpholin-4-yl)Me	1.323	0.269	-	0.533
**21**	Cl	OMe	H	(piperidin-1-yl)Me	1.245	0.339	-	0.915
**22**	Cl	OMe	H	(pyrrolidin-1-yl)Me	-0.096	0.473	-	0.452
**23**	Cl	OMe	H	(4-methylpiperazin-1-yl)Me	-0.477	0.510	-	0.014
**24**^b^	Cl	OMe	H	(diethylamino)Me	1.304	0.419	-	0.442
**25**	Cl	OMe	H	(dimethylamino)Me	0.298	0.258	-	0.322
**26**	Cl	OMe	H	[ethyl(methyl)amino]Me	1.120	0.238	-	0.267
**27**	Cl	OMe	H	[(2-hydroxyethyl)(methyl)amino]Me	-0.477	0.338	-	-0.233
**28**	Cl	OMe	H	{[2-(acetyloxy)ethyl](methyl)amino}Me	0.490	0.248	-	0.175
**29**	Cl	OMe	H	-[(2-methoxy-2-oxoethyl)(methyl)amino]Me	-0.477	0.272	-	0.222
**30**	Cl	OMe	H	-{[3-(dimethylamino)propyl](methyl)amino}Me	0.329	0.255	-	0.346
**31**	H	H	H	H	-1.301	-0.802	-1.479	-1.340
**32**^b^	H	H	(dimethylamino)Me	H	-1.301	-1.177	-1.516	-1.530
**33**	H	H	(pyrrolidin-1-yl)Me	H	-1.301	-1.255	-1.408	-1.401
**34**	H	H	(piperidin-1-yl)Me	H	-1.301	-1.248	-1.096	-1.049
**35**	H	H	(morpholin-4-yl)Me	H	-1.301	-1.372	-1.359	-1.363
**36**^b^	H	H	(thiomorpholin-4-yl)Me	H	-1.301	-1.314	-1.307	-1.381
**37**	H	H	(4-methylpiperazin-1-yl)Me	H	-1.301	-1.357	-1.264	-1.340

^a^Compounds **20**‒**30** are from reference [24], compounds **31**‒**37** are from reference [23].

^b^Compounds predicted in the test set.

### Docking

The binding modes of each compound into the active site of CDK1 were determined using ligand-receptor molecular docking; Glide software from Schrödinger suite was used [25]. This software has many successful aplications and is widely used for drug discovery [26,27], structure-activity relationship analysis [28–30], virtual screening [31,32], pharmacophore modeling [33–35], evaluation of enzymatic reaction pathways [36,37], and other studies.

Protein coordinates were extracted from the crystal structure of the complex CDK1-Cyclin bound to an ATP-competitive inhibitor (code 4Y72 in Protein Data Bank) [38]. Water molecules inside the active site were deleted before docking experiments to allow each compound to freely find its best pose inside the protein. A grid box of 28Å x 28Å x 28Å was centered on the center of mass of the inhibitor in this crystal structure covering the ATP-binding site of CDK1. To assign ionization states, ring conformations and stereochemistry of the compounds, the module LigPrep was used (LigPrep 3.0, Maestro 10.2.011, Schrödinger LLC). Docking parameters were used as in previous works [28–30]. Glide standard (SP) and extra-precision (XP) modes were used. From the found poses, the ones that showed the lower total docking energy (i.e. more favorable pose) were chosen.

### QSAR modeling

CoMFA and CoMSIA models were carried out to predict and interpret the compound biological activities. Dataset was divided in training and test sets (30 and 7 compounds respectively). Two alignment methods were used for QSAR applications: by considering a common structure as a template (self-consistent template alignments, TA in this text), and by considering the poses obtained by docking, under binding site constraints (X-ray-based alignment or docking alignment, DA in this text) [39].

CoMFA and CoMSIA were performed using Sybyl-X 1.1 software of Tripos suit [40]. Field descriptors were calculated on the tridimensional (3D) conformations obtained from both TA and DA alignments. Compounds contained in the training set were placed in a rectangular grid extending beyond 4 Å in each direction from the coordinates of each molecular structure. The interaction energies between a probe atom (sp^3^ hybridized carbon atom with +1 charge) and all compounds were computed at the surrounding points, using a volume-dependent lattice with 2.0 Å grid spacing. Then, standard Sybyl parameters were used for a partial least squares (PLS) analysis. The number of components in the PLS models was optimized by using Q^2^ value, obtained from the leave-one-out (LOO) cross-validation procedure, with the SAMPLS [41] sampling method. The number of components was increased until additional components did not increase Q^2^ by at least 5% per added component. The CoMFA models were generated by using steric and electrostatic probes with standard 30 kcal/mol cutoffs. In the CoMSIA analyses, similarity is expressed in terms of steric occupancy, electrostatic interactions, local hydrophobicity, and hydrogen bond (HB) donor and acceptor properties, using a 0.3 attenuation factor.

## Results and Discussion

### Docking results

There is no previous information about the binding poses of flavonoids inside CDK1 binding site; therefore, the quality of the obtained docking results was evaluated by analyzing the structural elements that typically are found in the complexes between PKs and inhibitors. It is known that ATP-competitive PK inhibitors generally mimic binding of the adenine of ATP to the hinge region of the PK; therefore, it is expected that the studied flavonoids have HB interactions with the hinge region residues.

**Fig 2:**
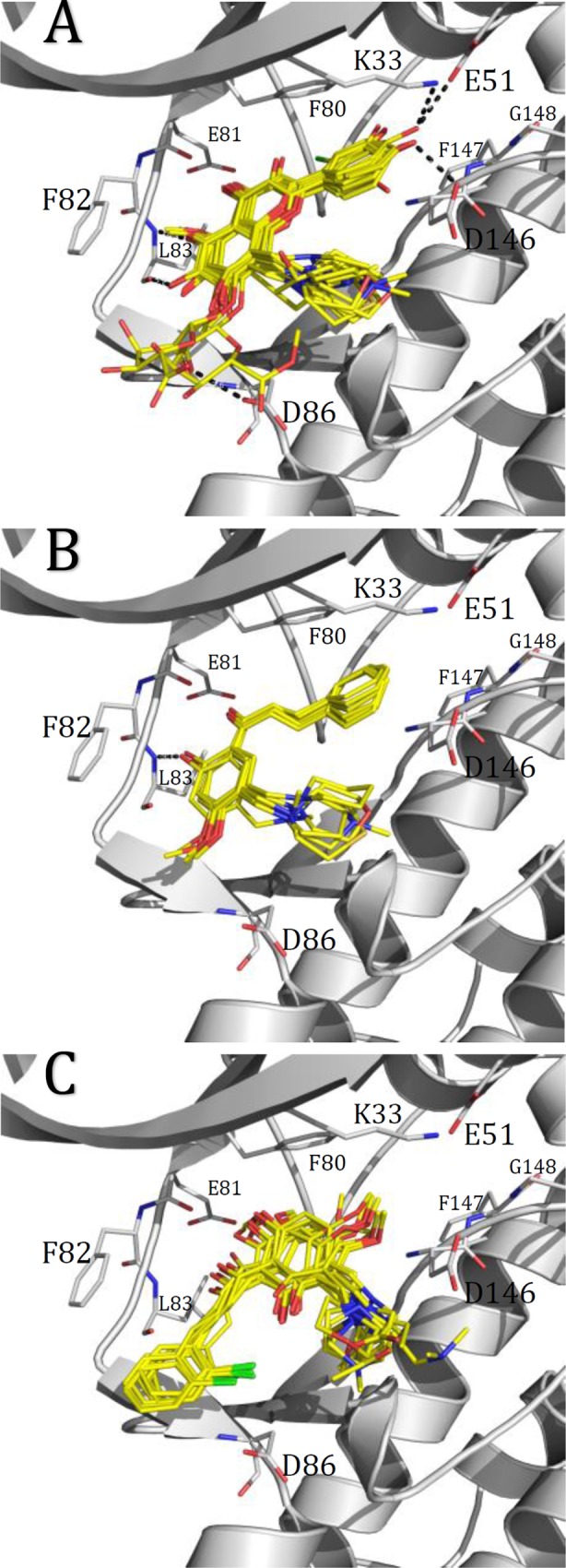
Binding modes of the compounds under study into CDK1 binding site. (A) flavones, compounds **1**–**19** (B) chalcones with orientation I, compounds **31**–**37**, (C) chalcones with orientation II, compounds **20**–**30**.

After completing docking experiments, we noticed that the compounds under study adopted two different orientations into the active site of CDK1 (orientations I and II in this manuscript, Fig 2). All the flavones **1**−**19** (including flavopiridol) and the poor active chalcones **31**−**37** adopted the orientation I. Flavones orientation I (Fig 2A) has B-ring turned to the C-helix and 4’-OH hydrogen-bonded to the side chain of Glu51 (the conserved glutamate of the C-helix), Lys33 (catalytic lysine) and/or Asp146 (DFG aspartate). Meanwhile, 6-OH of the A-ring is hydrogen-bonded to the backbone CO group of Leu83 of the hinge region. In addition, 5-OH or OMe groups of the A-ring form HB with the backbone NH group of Leu83 of the hinge region. Substituents at position 7 of the A-ring are solvent exposed and substituents at position 8 are in the pocket between the residues Asp146 (DFG), Gln132, Asn133, and Tyr15. Chalcones **31**−**37** have the same orientation (Fig 2B), but they do not form HB interactions with the CO of Leu83, and with the polar groups in the zone that contains the C-helix conserved glutamate, the catalytic lysine, and the DFG aspartate. The lack of polar substituents at B-ring should be the main factor responsible of poor activities of these compounds.

The chalcones **20**−**30** adopt the orientation II (Fig 2C), where the direction of B-ring opposes that of orientation I. B-ring in these chalcones is exposed to the solvent, while CO of the chalcone (at position 4 adopting flavones nomenclature) is near the backbone NH of Leu83 of the hinge region, and 5-OH is near the backbone CO of Glu81 at the hinge region. HB contacts are not observed using docking experiments, but the obtained conformations suggest that HB should be formed after relaxing protein structure (maybe using molecular dynamics). Finally, substituents at position 8 adopt the same position observed for orientation I.

There is no structure of flavopiridol crystalized inside CDK1, but there is one structure that contains flavopiridol inside CDK9 (PDB code: 3blr) [42]. The orientation and interactions of flavopiridol in CDK9 at the ATP-binding site is different with respect to the one reported here for flavones (orientation I), but it is similar to the orientation II identified here for chalcones **20**–**30**. Flavopiridol is almost entirely buried in CDK9 and forms hydrogen bonds (HBs) from the flavopiridol carbonyl oxygen and hydroxyl at position 5 to hinge residues Cys106 NH and Asp104 CO (long) and contacts between the piperidinyl group N1 at position 8 of the flavone to side chain of Phe30 (Tyr15 analogue in CDK1). B-ring is oriented to the pocket entrance near the residue Asp109 (Asp86 analogue in CDK1).

There are several reports that found that flavonoids have shown a variety of binding modes in PKs [43–45]. The analysis of other flavonoids bound to PKs show that there could be different poses for flavonoids in a PK. For instance, Yokoyama et al. obtained crystals of several flavonoids forming complexes with death-associated protein kinase 1 (DAPK1) and they found two distinctive binding directions of flavonoids in this PK (type A and B binding modes in the mentioned reference) [6]. In the binding modes of quercetin, luteolin and morin (type A binding mode, PDB codes 5auw, 5auu, and 5auy respectively), B-ring is turned to the C-helix and 4’-OH is hydrogen-bonded to the side chain of Glu64 (the conserved glutamate of the C-helix), whereas 7-OH of the A-ring is hydrogen-bonded to the backbone CO group of Glu94 and the NH group of Val96 of the hinge region. On the other hand, apigenin, kaempferol and genistein have distinctive binding modes in terms of the direction of the B-ring (type B binding mode, PDB codes 5auv, 5aux, and 5auz respectively). 4’-OH of the B-ring is hydrogen-bonded to the hinge region and 7-OH is hydrogen-bonded to Lys42 (catalytic lysine), that is, the binding direction of type B roughly opposes that of type A.

There are also reports that demonstrate that one flavonoid could have different orientations inside different PK binding sites. For instance, crystal structures of quercetin in different PKs have different orientations. The crystal structure of quercetin inside Pim-1 (PDB code: 2o3p) shows two conformations; in both of them 3-OH of the C-ring is hydrogen-bonded to the backbone CO group of hinge region Glu121 and 5-OH of the A-ring forms a long HB with the backbone CO group of hinge region Pro123 [46]. One conformation orients B-ring to the DFG motif with 3’-OH and 4’-OH forming HBs with the side chain of Asp186 (DFG aspartate), and the other conformation orients B-ring to the catalytic lysine (Lys67) with 4’-OH forming HB with the side chain of this residue. On the other hand, the crystal structure of quercetin inside the tyrosine PK HCK (PDB code: 2hck) shows other orientation [47]. 5-OH of the A-ring is hydrogen-bonded to the backbone NH group of hinge region Met341 and 7-OH of the A-ring is hydrogen-bonded to the backbone CO group of hinge region Thr338. Meanwhile, B-ring is at the entrance of the pocket and is near the residue Ser345.

Significant differences are found when comparing the orientations and interactions of quercetin inside DAPK1, Pim-1 and HCK ATP binding sites (PDB codes 5auw, 2o3p and 2hck respectively). These evidences confirm that flavonoids, due to the large number of functional groups, could have different orientations inside different PK binding sites. Therefore, it is not unexpected that flavopiridol and its derivatives have different orientations and interactions inside CDK1 with respect to the interactions found in CDK9.

### QSAR models

In addition to docking experiments, we also build CoMFA and CoMSIA models in order to identify the structural features of the flavonoids that affect their inhibitory activities against CDK1. The models were developed using template alignment (TA models) and docking alignment (DA models). For both alignment rules, models were derived from different combinations of up to two fields for CoMFA and up to five fields for CoMSIA. The best models were selected by considering the statistical performance of the internal LOO cross-validation (Q^2^ > 0.5).

**Fig 3:**
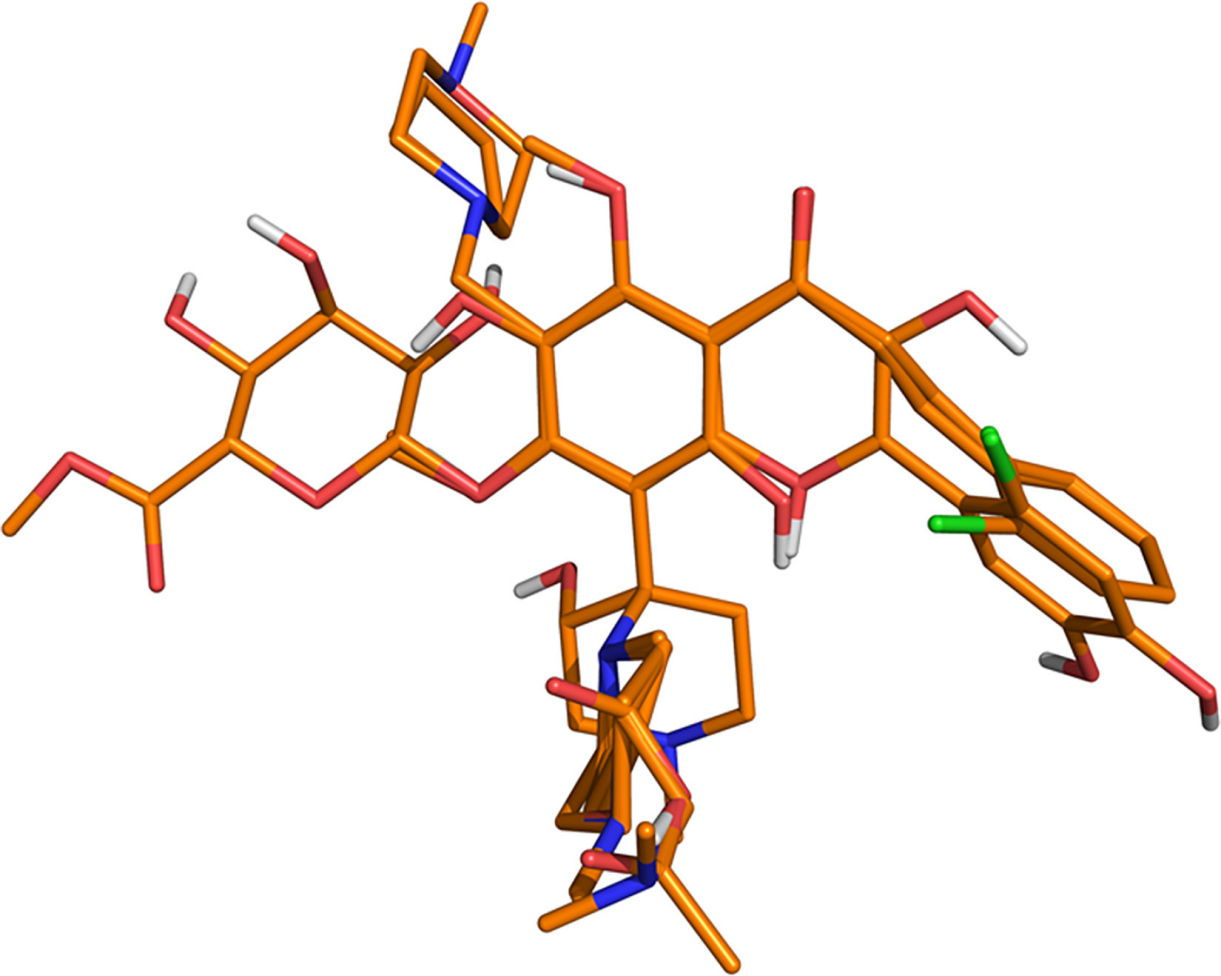
Template alignment (TA) for all compounds used for CoMFA and CoMSIA.

**Fig 4:**
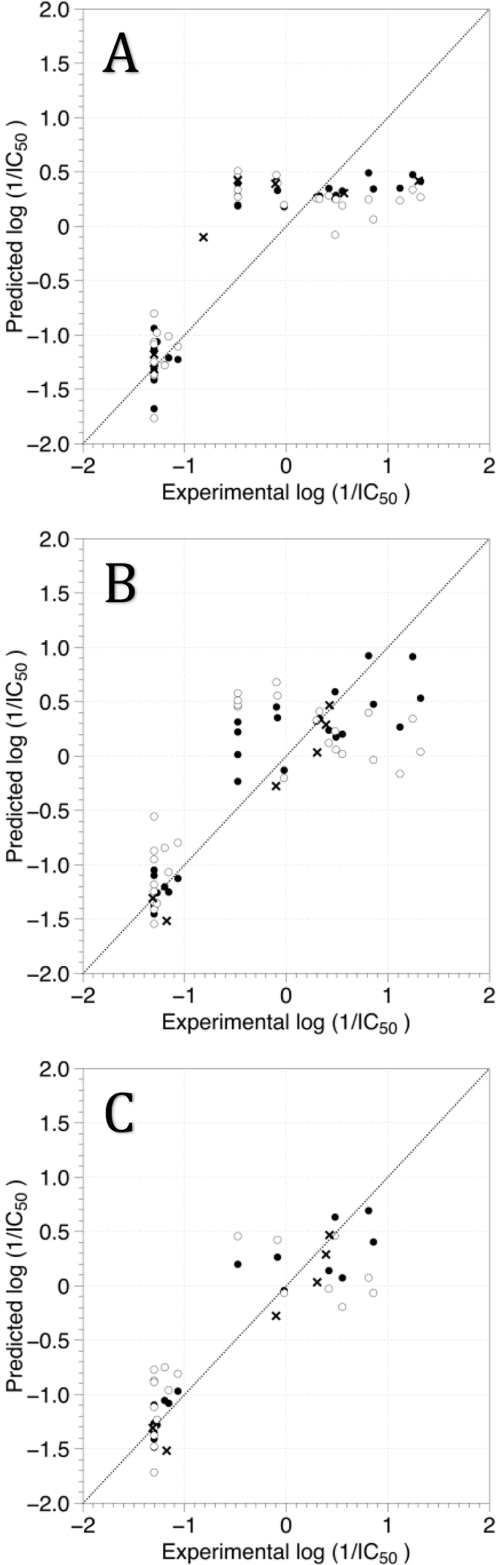
**Scatter plot of the experimental activities versus predicted activities for the best CoMSIA models using TA (A), DA (B) and DAI (C).** (●) training-set predictions, (○) LOO cross-validated predictions, (×) test-set predictions.

For TA, all the flavones and chalcones under study were aligned by atom-by-atom least-square fit. Fig 3 shows the TA of the molecules within the grid box used to perform the CoMFA and CoMSIA calculations. The results are presented in Table 3. The TA CoMFA models that used only the steric field or the electrostatic field (models CoMFA-S and CoMFA-E) were statistically adequate (Q^2^ > 0.5), but combination of both fields deteriorates the modeling. Better results were found for CoMSIA modeling when hydrophobic and donor HB fields were combined (Q^2^ = 0.647). The TA model CoMSIA-HD used three components and has hydrophobic and HB donor contributions of 49.2% and 50.8% respectively. The best TA model also performed successful predictions for the test set compounds; Fig 4A shows the plots for the calculated log(1/IC_50_) values derived for training set, LOO cross-validation process, and test set using this model.

**Table 3:** CoMSIA and CoMFA results using the best field combinations^a^.

Method	Model	TA			DA			DAI		
		NC	Q^2^	S_sv_	NC	Q^2^	S_sv_	NC	Q^2^	S_sv_
CoMFA	S	3	0.541	0.654	1	0.166	0.849	10	-0.032	1.245
	E	6	0.544	0.693	3	0.168	0.88	1	-0.088	0.904
	SE	4	0.484	0.707	7	0.213	0.931	5	0.136	0.914
CoMSIA	S	3	0.443	0.72	1	0.154	0.856	6	0.493	0.726
	E	2	0.334	0.773	2	0.236	0.828	3	0.239	0.802
	H	3	0.609	0.604	3	0.486	0.692	6	0.642	0.61
	D	4	0.634	0.596	3	0.288	0.814	2	0.071	0.86
	A	4	0.378	0.776	1	0.205	0.829	6	0.112	0.961
	HD	**3**	**0.647**	**0.574**	**4**	**0.514**	**0.687**	4	0.654	0.559
	S	3	0.613	0.601	3	0.416	0.738	4	0.655	0.558
	SD	3	0.626	0.59	2	0.307	0.789	2	0.194	0.801
	SHD	2	0.625	0.58	4	0.475	0.713	**3**	**0.669**	**0.529**

^a^NC is the number of components from the PLS analysis; Q^2^ and S_cv_ are the correlation coefficient and standard deviation, respectively, of the leave-one-out (LOO) cross-validation. The best models are in boldface. All the possible combinations were proved, but the best of all were reported here.

We also developed CoMFA and CoMSIA models encompassing the whole dataset using DA conformations for identifying the relevant chemical features which define the potency of the flavonoids as CDK1 inhibitors. The main difference with respect to previous models is that alignment is constrained by a binding site and chalcones are not superposed by chemical similitude because their different orientations I and II. The CoMFA models applied on DA conformations considering the whole dataset (DA models) were statistically unacceptable (Q^2^ < 0.5). However, better results were found for CoMSIA modeling when hydrophobic and donor HB fields were combined (Q^2^ = 0.514). Interestingly, the same fields can be used to explain the structure-activity relationship using TA and DA alignments.

The DA model CoMSIA-HD has a hydrophobic contribution of 53.8% and donor HB contribution of 46.2%. The best DA model also performed successful predictions for the test set compounds; Fig 4B shows the plots for the calculated log(1/IC_50_) values derived for training set, LOO cross-validation process, and test set using this model.

We also tried to construct models for describing only compounds with orientation I (DAI models) and models for describing only compounds with orientation II (DAII models). The results are presented in Table 3. No predictive DAII models were obtained, which probably occurred due to insufficient data (only 11 compounds with orientation II). However, predictive DAI models were obtained using CoMSIA applied to a training set of 20 compounds. The best model also included hydrophobic and HB donor fields, but with addition of the steric field (Q^2^ = 0.669). The contributions were 49.0%, 41.3%, and 9.7% for hydrophobic, HB donor, and steric fields respectively; it is noteworthy that the steric field has a negligible contribution. Fig 4C shows the plots for the calculated log(1/IC_50_) values derived for training set, LOO cross-validation process, and test set (6 compounds) using the best DAI model.

The above mentioned results suggest that the structure-activity relationship of the studied compounds can be explained considering hydrophobic and HB donor fields, but the steric field detects some minimal particularities of the orientation I. The correlation plots between the calculated and predicted activities (log (1/IC_50_) using the best CoMSIA models with TA, DA and DAI alignments in Fig 4 show that these models discriminate the more active inhibitors with respect to the less active ones.

**Fig 5:**
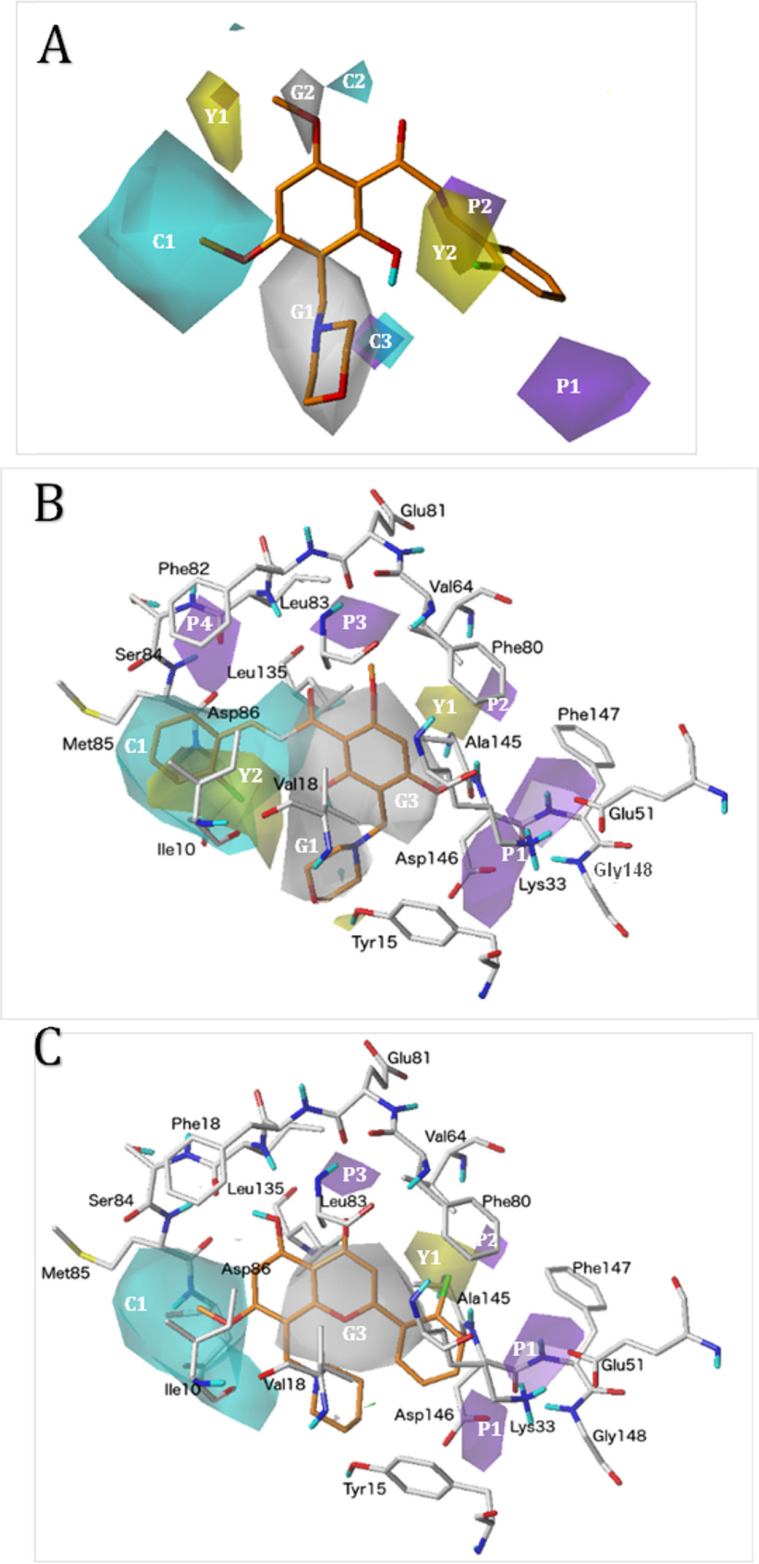
**CoMSIA contour maps for CDK1 inhibitors deriving from the best models for the TA (A), DA (B) and DAI (C) alignments.** Compound **20** is shown inside the fields in (A) and (B), and compound **4** is shown inside the fields in (C). In (B) and (C), the amino acid residues located close to the binding pocket of CDK1 are represented for comparing their position with the position of isopleths derived from the model. Hydrophobic field: yellow isopleths indicate regions where hydrophobic groups favor the activity, and gray isopleths indicate regions where hydrophilic groups favor the activity. HB donor field: cyan isopleths indicate regions where HB donors favor the activity, and purple isopleths indicate regions where HB donors disfavor the activity. Steric field (in C): green isopleths indicates region where bulky groups favor the activity.

The contour plots of the best CoMSIA models for TA, DA and DAI alignments are presented in Fig 5. For simplicity, the interactions between only the most active CDK1 inhibitor (compound **20**) and the contour maps derived from TA and DA alignments are shown (Fig 5A and 5B); the interactions between the most active CDK1 inhibitor of the DAI subgroup (compound **4**) and the contour maps derived from DAI alignment are also shown (Fig 5C). Isopleths in Fig 5 represent the volumes of regions where chemical groups have positive or negative effects on CDK1 inhibitory activity. Yellow and gray colors illustrate the zones where hydrophobic field has positive and negative effects respectively; meanwhile, cyan and purple colors illustrate the zones where HB donor field has positive and negative effects respectively.

Hydrophobic and HB donor CoMSIA isopleths are presented in Fig 5A for the best model using TA alignment. The highly active compound **20** is displayed in the maps as a template. A yellow contour at position 6 of the flavonoids (Y1 in Fig 5A) indicates that hydrophilic substituents are not the best option at this position. In fact, the less active compounds **32**–**37** have hydrophilic groups at this position. Other yellow contour is in the surroundings of the B ring (Y2 in Fig 5A) which indicates that hydrophobic groups are the best option in this zone. In fact, the presence of 2-Cl substituents at B ring seems to be adequate for having good CDK1 inhibitors; however, analogous compounds with OH substituents are less active (compounds **14**–**18** have log(1/IC_50_) values below -1.1). A big gray contour at position 8 of the A ring (G1 in Fig 5A) indicates that hydrophilic groups are desired at this position. In fact, the most active compounds **20**, **21**, and **24** (log(1/IC_50_) values above 1.2) have hydrophilic groups in this zone. Other gray contour is at position 5 of the A ring (G2 in Fig 5A) indicating that a hydrophilic group is essential at this position. OH or OMe substituents are at position 5 of the A ring for almost all the compounds, but the less active compounds **31**–**37** lack of oxygen-containing groups at this position.

Cyan and purple isopleths in Fig 5A represent regions where HB donors favored and disfavored the activity, respectively. A big cyan contour at position 7 of the A ring (C1 in Fig 5A) indicates that HB donors are preferred instead of OMe groups. The other small cyan contours (C2 and C3 in Fig 5A) suggest that HB donors are tolerated at positions 5 and 8 of the A ring. A purple contour at the surroundings of the B ring (P1 in Fig 5A) indicates that HB donors are not desired in this zone. In fact, compounds with OH substituents at the B ring are poorly active (compounds **14**–**18**). Other purple contour is at position 3 of the C ring of flavones (P2 in Fig 5A) indicating that HB donors are not desired at this position. In fact, the presence of an OH group at this position deteriorates the CDK1 inhibitory activity (compounds **14**–**18**).

The hydrophobic and HB donor CoMSIA isopleths for the best model using DA alignment are presented in Fig 5B. The highly active compound **20** is displayed in the maps as a template compound and the superposition of CoMSIA contour plots on CDK1 active-site residues is also shown.

It has been previously described that the applications of 3D-QSAR methodologies by using template and docking alignments usually lead to different models containing different fields [48]. However, we found that the above described contours Y1, Y2, G1, C1, P1, and P2 are also present in our best DA CoMSIA model (Fig 5B). Therefore, we can relate these contours with the surrounding amino acids in the CDK1 ATP binding site. It is important to remember that A ring of the flavonoids is superposed for all the compounds when TA alignment is used, but A ring is not superposed in DA alignment for compounds that have orientations I and II; i.e., A ring for compounds with orientation I is in a different 3D position with respect to that for compounds with orientation II (Fig 2). Below, we describe the binding site amino acids related to the identified contours:

Y1: Close to Phe80 (gatekeeper residue). It is at position 6 of chalcones with orientation II (as in TA), but it is at position 3 of flavones and chalcones with orientation I.

Y2: Close to Ile10. It is in the surroundings of the B ring of chalcones with orientation II (as in TA), but it is near position 7 of flavones and chalcones with orientation I.

G1: Close to Tyr15. It is at position 8 for both orientations (as in TA).

C1: Around the residues Ile10, Met85, Val18, and Asp86. It is in front of the position 7 of the flavones and chalcones with orientation I (as in TA), but it is in the zone of the B ring of chalcones with orientation II.

P1: Close to the residues Lys33 (catalytic lysine), Glu51 (C-helix), and Asp146 (DFG). It is in the zone of the B ring of the flavones and chalcones with orientation I (as in TA), but it is in front of the position 7 of the chalcones with orientation II.

P2: Close to Phe80 (gatekeeper residue). It is at position 3 of flavones and chalcones with orientation I (as in TA), but it is at position 6 of chalcones with orientation II.

Two additional purple contours near the hinge region (P3 and P4) indicate that HB donors are not required in these zones, and an additional gray contour (G3) is located in the zone of the C ring for compounds with orientation I and the zone of the A ring for compounds with orientation II. The contours Y1, Y2, G1, C1, P1, and P2 in DA can be interpreted as in TA, but their interactions with the amino acids of the ATP binding site allow explaining the chemical features relevant for the structure-activity relationship. Y1 and P2 define the requirements of the flavonoids for their interactions with the CDK1 gatekeeper residue Phe80: hydrophobic groups and no HB donors for substituents at positions 3 or 6 of the flavonoids are the requirements for interaction with this residue. Y2 and C1 define the requirements of the flavonoids for their interaction with the solvent exposed residues of the ATP binding site: hydrophobic groups on the B ring or HB donors at the position 7 of the flavonoids are the requirements for interactions in this zone. G1 defines that hydrophilic substituents at position 8 of the flavonoids are essential for establishing interactions inside the pocket near Tyr15. Finally, P1 indicates that HB donors at the B ring are not desired. These groups can form HB interactions with the catalytic lysine, the C-helix glutamate, and the DFG aspartate, but these interactions deteriorate the CDK1 inhibitory activity.

The hydrophobic, HB donor and steric CoMSIA isopleths for the best model using DAI alignment are presented in Fig 5C. The highly active compound **4** is displayed in the maps as a template and the superposition of CoMSIA contour plots on CDK1 active-site residues is also shown. The contours Y1, C1, P1, P2, G3, and P3 are also present in our best DA1 CoMSIA model (Fig 5C). It is assumed that their interpretation is the same that was described for DA CoMSIA model. The steric field, with a negligible contribution (only 9.7%) is represented by a very small green isopleth near the substituent at position 8 of the flavonoids (Fig 5B). Because the very small size of this isopleth, we consider that its influence to the structure-activity relationship is minimal.

## Conclusions

The structural requirements of flavonoids derivates (flavones and chalcones) as CDK1 inhibitors were studied by using docking and 3D-QSAR methods applied on docked aligned (DA) and template aligned (TA) structures. Some characteristics of these compounds that explain their differential activities were described such as their orientation and the interactions that they establish with the residues located in the CDK1 ATP binding site.

Docking experiments led to orientation I for flavones and some chalcones and orientation II for the remaining chalcones. In previous literature, series of flavonoids that have a variety of binding modes in a PK binding site have been commonly observed.

The most interesting finding in the QSAR analysis is that mainly HB interactions as well as hydrophobic interactions are the key factors that influence the potency of flavonoid derivatives as CDK1 inhibitors. Interestingly, the same chemical features were identified for DA and TA alignments. The structural map of the properties that are desired for highly active compounds and their relation with the residues of the CDK1 active site were identified.
